# Historical Biogeography of endemic seed plant genera in the Caribbean: Did GAARlandia play a role?

**DOI:** 10.1002/ece3.3521

**Published:** 2017-10-24

**Authors:** María Esther Nieto‐Blázquez, Alexandre Antonelli, Julissa Roncal

**Affiliations:** ^1^ Department of Biology Memorial University of Newfoundland St. John's NL Canada; ^2^ Department of Biological and Environmental Sciences University of Göteborg Göteborg Sweden; ^3^ Gothenburg Botanical Garden Göteborg Sweden; ^4^ Gothenburg Global Biodiversity Centre Göteborg Sweden

**Keywords:** Antilles, Cenozoic, colonization, dispersal, island biogeography, West Indies

## Abstract

The Caribbean archipelago is a region with an extremely complex geological history and an outstanding plant diversity with high levels of endemism. The aim of this study was to better understand the historical assembly and evolution of endemic seed plant genera in the Caribbean, by first determining divergence times of endemic genera to test whether the hypothesized Greater Antilles and Aves Ridge (GAARlandia) land bridge played a role in the archipelago colonization and second by testing South America as the main colonization source as expected by the position of landmasses and recent evidence of an asymmetrical biotic interchange. We reconstructed a dated molecular phylogenetic tree for 625 seed plants including 32 Caribbean endemic genera using Bayesian inference and ten calibrations. To estimate the geographic range of the ancestors of endemic genera, we performed a model selection between a null and two complex biogeographic models that included timeframes based on geological information, dispersal probabilities, and directionality among regions. Crown ages for endemic genera ranged from Early Eocene (53.1 Ma) to Late Pliocene (3.4 Ma). Confidence intervals for divergence times (crown and/or stem ages) of 22 endemic genera occurred within the GAARlandia time frame. Contrary to expectations, the Antilles appears as the main ancestral area for endemic seed plant genera and only five genera had a South American origin. In contrast to patterns shown for vertebrates and other organisms and based on our sampling, we conclude that GAARlandia did not act as a colonization route for plants between South America and the Antilles. Further studies on Caribbean plant dispersal at the species and population levels will be required to reveal finer‐scale biogeographic patterns and mechanisms.

## 1 INTRODUCTION

Although islands cover only about 5% of the Earth's surface, they contain about a quarter of all terrestrial plant species (Caujapé‐Castells, 2011). Explaining the high biological diversity and endemicity of islands has been a topic of study in the last three centuries, pioneered by Darwin (1876) and Wallace (1892). The structure of insular communities is the result of the interaction among three fundamental biological processes: immigration, speciation, and extinction (Lomolino, Riddle, & Whittaker, 2017; Whittaker, Fernández‐Palacios, Matthews, Borregaard, & Triantis, 2017; Whittaker, Triantis, & Ladle, 2008). According to the new synthesis in island biogeography theory (Lomolino et al., 2017), these three fundamental processes are scale dependent affecting different levels of biological organization from individuals, to populations or communities, and biotas. Evolutionary and geological dynamics have been identified to affect the biotic level of insular organization (Haila, 1990).

The Caribbean archipelago (i.e., Greater and Lesser Antilles, and the Bahamas) is one of the world's 34 biodiversity hotspots (Mittermeier et al., 2004) and represents the most important insular system in the Neotropics (Maunder et al., 2008). Despite the relatively small land area of this archipelago, there are nearly 13,000 seed plant species, of which almost 8,000 are endemic (Acevedo‐Rodríguez & Strong, 2008). This alpha‐diversity is similar to that of Madagascar, and three times larger than that of New Caledonia (Myers, Mittermeier, Mittermeier, Fonseca, & Kent, 2000). There are 180 seed plant genera endemic to the Caribbean (Francisco‐Ortega et al., 2007) which represents 13.2% of the total number of genera on the islands, and 86 of the 180 endemic genera (47.7%) are monotypic. Endemic genera are concentrated in the Greater Antilles, especially in Cuba and Hispaniola, the largest and most heterogeneous islands (Santiago‐Valentín & Olmstead, 2004).

The vast flora diversity in the Caribbean can be explained not only by its proximity to the American continent, which might have facilitated successful dispersal from an outstandingly rich biota, but also by the very complex interaction of geological events, which include volcanism, plate tectonic movements, and intervals of island emergence and submergence (Graham, 2003; Iturralde‐Vinent & MacPhee, 1999). Moreover, climatic change, through cooling or warming periods (Zachos, Pagani, Sloan, Thomas, & Billups, 2001), has greatly influenced the region since the Cretaceous (Fritsch & McDowell, 2003) and has had an impact on major sea‐level changes. These sea‐level changes had in turn an effect on the connectivity between the continent and the islands, creating further migration opportunities (Weigelt, Steinbauer, Cabral, & Kreft, 2016). The geological history of the Greater and Lesser Antilles are quite distinct from one another, and the main sequence of events is described in detail in Graham (2003). The Greater Antilles originated in the Cretaceous [*c*. 130 Million years ago (Ma)], forming a volcanic chain of sea mountains between North and South America (Pindell & Kennan, 2009). This chain of islands, known as Proto‐Antilles, moved northeastward until they collided first with the Yucatan Peninsula (*c*. 84 Ma) and then with the Bahamas Platform in the Early Eocene (*c*. 56 Ma). The Lesser Antilles were formed subsequently between the Middle Eocene (*c*. 47–38 Ma, in the north) and the Oligocene (*c*. 34–23 Ma, in the south) as a result of the subduction of the South American Plate under the Caribbean Plate. By the Middle Eocene (*c*. 49 Ma), most of the Greater and Lesser Antilles were above water. This geological activity for the last 100 Ma (Burke, 1988) might have presented significant opportunities for speciation, colonization, and vicariance (Hedges, 2001).

In 1999, Iturralde‐Vinent and McPhee introduced a controversial hypothesis, the “GAARlandia (Greater Antilles + Aves Ridge) land bridge.” They proposed that colonization of the Antilles was possible from northeast South America through a quasi‐continuous land bridge or island chain that lasted for a period of 1–2 Ma, close to the Eocene–Oligocene boundary, *c*. 34 Ma. The Eocene–Oligocene boundary coincides with a major drop in temperature and sea level that might have affected connectivity between regions exposing land areas (Hedges, 2001). Studies that support the colonization role of GAARlandia are primarily based on molecular dating estimates, and comprise amphibians (Alonso, Crawford, & Bermingham, 2012), invertebrates (Binford et al., 2008), vertebrates (Hulsey, Keck, & Hollingsworth, 2011), and also plants, as shown for the genus *Styrax* (Styracaceae, Fritsch, 2003), *Moacroton* (Euphorbiaceae, van Ee, Berry, Riina, & Gutiérrez Amaro, 2008), and *Copernicia* (Arecaceae, Bacon, Baker, & Simmons, 2012). Despite this evidence, the existence of GAARlandia is still a debatable hypothesis to explain lineage colonization and diversification in the Caribbean (Ali, 2012), due to limited geological and paleoceanographical evidence supporting its existence and because molecular and biogeographic evidence is still incomplete for the Caribbean biota.

While floristic studies have shown strong links between the Caribbean flora and that of the surrounding continental landmasses (Acevedo‐Rodríguez & Strong, 2008), little is known regarding the precise timing and geographic origin of the flora as a whole. Most insight on Caribbean historical biogeography results from molecular phylogenies of vertebrates (Dávalos, 2004; Hedges, 2006; Hulsey et al., 2011; Monceau, Cezilly, Moreau, Motreuil, & Wattier, 2013), which suggest a combination of dispersal and vicariance for the Antillean fauna. North (NA) and Central America (CA) have been identified as colonization sources for active dispersers, such as birds, bats, and freshwater fishes (Hedges, 1996, 2006) into the Caribbean region. In contrast, South America (SA) has been suggested as the main source for passive dispersers (nonvolant fauna), which would require floating mechanisms (Hedges, 1996, 2006), and for vertebrates using potential land bridges for island colonization (Alonso et al., 2012; Dávalos, 2004).

Francisco‐Ortega et al. (2007) provided a checklist of Caribbean endemic seed plant genera and a review of molecular phylogenetic studies of these plants. Their review highlighted that DNA phylogenies were available for only 35% of the Antillean genera. Since then, several molecular phylogenies that include Caribbean endemic genera have been published (e.g., Appelhans, Keßler, Smets, Razafimandimbison, & Janssens, 2012; Jestrow, Rodríguez, & Francisco‐Ortega, 2010; Jestrow, Valdés, et al., 2012), revealing a complex biogeographic history (Roncal, Zona, & Lewis, 2008). Some endemic genera have sister taxa that are widely distributed in continental America (Lavin, Wojciechowski, Gasson, Hughes, & Wheeler, 2003; Lavin, Wojciechowski, et al., 2001; Rova, Delprete, Andersson, & Albert, 2002; Wurdack, Hoffmann, & Chase, 2005), others have relatives with a more restricted continental distribution (Lavin, Pennington, et al., 2001; Baldwin et al., 2002; Wojciechowski, Lavin, & Sanderson, 2004), and a few are sister to taxa that are native to regions outside the Neotropics, such as Africa (Lavin, Pennington, et al., 2001; Lavin, Wojciechowski, et al., 2001), Polynesia (Kimball & Crawford, 2004), and New Caledonia (Motley, Wurdack, & Delprete, 2005).

With the aim of providing insights into the origin and evolution of the Caribbean flora, we targeted endemic seed plant genera. We focused on genera because most plant phylogenies are still poorly sampled at the species level, rendering the inference of range evolution problematic and biased by the inclusion of common, widespread species with island and continental distributions, and fewer island endemics. It was also beyond the scope of this study to analyze the biogeographic history of individual endemic species within nonendemic genera. Even though higher taxa (e.g., genera and families) may not be as intercomparable as biological species, processes normally considered in the context of speciation like divergent selection and geographic isolation can generate evolutionary significant units above the species level (Barraclough, 2010; Barraclough & Humphreys, 2015). Plant genera can therefore also be used as units of biodiversity.

We reconstructed a dated phylogenetic tree and tested different biogeographic scenarios to address the following questions: (1) When did endemic seed plant genera diverge from their sister taxa, and (2) what were the most likely regions that ancestors of endemic genera occupied? Our hypotheses are (1) GAARlandia played a major role as a migration route in the colonization of the Caribbean Islands. Under this hypothesis, we expect to find the origin of endemic genera (i.e., mean stem to crown ages) contemporaneous with the hypothesized presence of GAARlandia. (2) Endemic genera descended from South American ancestors because of their proximity to GAARlandia, which facilitated colonization from SA more than from CA or NA, and considering the asymmetry in dispersal or migration directionality during large part of the Neogene observed in birds, plants and mammals (Bacon, Silvestro, Jaramillo, Tilston, & Chakrabarty, 2015; Weir, Bermingham, & Schluter, 2009). Through a taxon sampling of 32 endemic seed plant genera, this study provides a comprehensive evolutionary and biogeographic framework to understand the historical assembly of the Caribbean flora.

## 2 MATERIAL AND METHODS

### 2.1 Taxon sampling selection

We searched for sequences from all endemic plant genera following the compilation by Francisco‐Ortega et al. (2007) on the data matrix of Zanne et al. (2014) who reconstructed a dated phylogeny for 32,223 plant species. Zanne et al. (2014) used the International Plant Names Index (IPNI), Tropicos, The Plant List and Angiosperm Phylogeny Group (APG) to verify taxonomic nomenclature. We found 56 species within 41 endemic genera in Zanne et al. (2014). Of these 41 endemic genera, 33 are included in the 35% of Caribbean endemic genera included in molecular phylogenies stated by Francisco‐Ortega et al. (2007). Therefore, we have included 52% endemic genera for which there were molecular phylogenies available at the time of the publication. We used the NCBI taxonomy facility (Federhen, 2012) to select up to 10 species for every genus within the suprageneric rank to which the endemic genera belong. When genera contained more than 10 species, we selected species that represented the entire distributional range of the genus, and with complete sequences available in the Zanne et al. (2014) matrix.

### 2.2 DNA sequence selection and alignment

Of the seven gene regions available in Zanne et al. (2014), we selected four (18S rDNA*, atpB, matK,* and *rbcL*) for our alignment. We excluded the 26S rDNA region because it was not well represented (only 17 sequences were available for our taxon sampling). The ITS and *trnL‐trnF* gene regions were available for a fair number of species (511 and 594, respectively) but were also excluded because sequences were difficult to align. Each of the four‐gene regions was aligned independently using MAFFT v. 7.187 on XSEDE (Katoh & Standley, 2013) via the CIPRES Science Gateway (Miller, Pfeiffer, & Terri Schwartz, 2010). Manual trimming and concatenation of gene regions were performed in GENEIOUS v. 7.1.9 (Kearse et al., 2012). Our final four‐gene concatenated matrix had a total length of 5,462 bp and contained 625 seed plant species (Spermatophyta) within 319 genera in 20 families, including 41 Caribbean endemic genera (Table 1). We had 37% missing nucleotide data in this final alignment.

**Table 1:** Seed plant genera endemic to the Caribbean Islands sampled in this study. *Endemic genus sampling* indicates the number of species sampled in this study divided by the total number of species (based on Francisco‐Ortega et al., 2007); *suprageneric sampling* indicates the number of genera in a suprageneric taxon sampled in this study divided by the total number of genera; *suprageneric rank* refers to the name of suprageneric rank and in parenthesis the number of species within this taxonomic rank included in this study. NCBI Taxonomy facility (Federhen, 2012) was used to select up to 10 species for every genus within their suprageneric rank to which the endemic genera belong to

Endemic genus	Endemic genus sampling	Suprageneric sampling	Suprageneric rank (number of species)	Family
*Acidocroton*	1/3	7/10	Tribe Crotoneae (16)	Euphorbiaceae
*Acidoton*	1/8	6/12	Tribe Plukenetieae (9)	Euphorbiaceae
*Anacaona*	1/1	12/13	Tribe Cucurbiteae (20)	Cucurbitaceae
*Arcoa*	1/1	46/56	Tribe Caesalpinieae (79)	Fabaceae
*Bonania*	1/8	20/23	Tribe Hippomaneae (30)	Euphorbiaceae
*Broughtonia*	4/6	50/54	Subtribe Laeliinae (117)	Orchidaceae
*Brya*	1/4	30/48	Tribe Dalbergieae (53)	Fabaceae
*Calycogonium*	1/36	3/19–23	Tribe Miconieae (13)	Melastomataceae
*Chascotheca*	1/2	3/3	Subtribe Astrocasiinae (5)	Phyllanthaceae
*Cubanola*	1/2	9/28	Tribe Chiococceae (19)	Rubiaceae
*Dendropemon*	1/36	4/9	Subtribe Psittacanthinae (4)	Loranthaceae
*Dilomilis*	1/5	50/54	Subtribe Laeliinae (117)	Orchidaceae
*Ditta*	1/2	7/7	Tribe Adenoclineae (9)	Euphorbiaceae
*Doerpfeldia*	1/1	1/1	Tribe Doerpfeldieae (1)	Rhamnaceae
*Domingoa*	2/3	50/54	Subtribe Laeliinae (117)	Orchidaceae
*Espadaea*	1/1	2/4	Subfamily Goetzeoideae (2)	Solanaceae
*Fuertesia*	1/1	2/4	Subfamily Gronovioideae (2)	Loasaceae
*Goetzea*	1/2	2/4	Subfamily Goetzeoideae (2)	Solanaceae
*Grimmeodendron*	1/2	20/23	Tribe Hippomaneae (30)	Euphorbiaceae
*Haenianthus*	1/2	15/18	Tribe Oleeae (53)	Oleaceae
*Hebestigma*	1/1	10/13	Tribe Robinieae (23)	Fabaceae
*Hemithrinax*	3/3	9/10	Tribe Cryosophileae (14)	Arecaceae
*Lasiocroton*	3/5	5/6	Tribe Adelieae (18)	Euphorbiaceae
*Leptocereus*	1/12	14/27	Tribe Echinocereeae (22)	Cactaceae
*Leucocroton*	3/28	5/6	Tribe Adelieae (18)	Euphorbiaceae
*Microcycas*	1/1	2/8	Family Zamiaceae (6)	Zamiaceae
*Moacroton*	1/8	7/10	Tribe Crotoneae (16)	Euphorbiaceae
*Neobracea*	3/8	2/2	Subtribe Pachypodiinae (7)	Apocynaceae
*Neocogniauxia*	1/2	50/54	Subtribe Laeliinae (117)	Orchidaceae
*Penelopeia*	1/1	12/13	Tribe Cucurbiteae (20)	Cucurbitaceae
*Petitia*	1/2	2/7	Subfamily Viticoideae (2)	Lamiaceae
*Picrodendron*	1/1	13/19	Family Picrodendraceae (13)	Picrodendraceae
*Pictetia*	1/8	30/48	Tribe Dalbergieae (53)	Fabaceae
*Poitea*	3/12	10/13	Tribe Robinieae (23)	Fabaceae
*Psychilis*	2/15	50/54	Subtribe Laeliinae (117)	Orchidaceae
*Quisqueya*	1/4	50/54	Subtribe Laeliinae (117)	Orchidaceae
*Rhodopis*	1/2	47/84	Tribe Phaseoleae (86)	Fabaceae
*Stahlia*	1/1	46/56	Tribe Caesalpinieae (79)	Fabaceae
*Synapsis*	1/1	2/3	Family Schlegeliaceae (2)	Schlegeliaceae
*Tetramicra*	1/13	50/54	Subtribe Laeliinae (117)	Orchidaceae
*Zombia*	1/1	9/10	Tribe Cryosophileae (14)	Arecaceae

### 2.3 Phylogenetic reconstruction and dating

We performed tree searches using the four‐gene concatenated matrix under a maximum likelihood (ML) approach. Phylogeny reconstruction was performed on RAxML‐HPC2 version 8.2.8 (Stamatakis, 2006) via the CIPRES Science Gateway using the rapid bootstrap algorithm with 500 replicates. We selected six gymnosperms in the Zamiaceae family to root the tree, which included the monotypic endemic Caribbean genus *Microcycas* and five *Zamia* species. We used JMODELTEST2 v.0.1.1 (Darriba, Taboada, Doallo, & Posada, 2012) via the CIPRES Science Gateway to select the best nucleotide substitution model for the four‐gene alignment under the Akaike information criterion (AIC, Akaike, 1974). The best‐fit model was GTR + I  +  Γ, which was selected for subsequent analyses.

In order to estimate absolute divergence times, we inferred a time‐calibrated phylogenetic tree using a Bayesian inference (BI) approach as implemented in BEAST 2.3.1 (Bouckaert et al., 2014). Analysis on the concatenated matrix used the uncorrelated lognormal (UCLN) relaxed clock (Drummond, Ho, Phillips, & Rambaut, 2006). The tree prior was set to the Yule model, which models a constant lineage birth rate for each branch in the tree. Ten calibration points were applied to the dating analysis. In order to avoid overestimation of divergence ages, we chose the oldest fossil found to constrain the stem of each particular clade (Table 2).

**Table 2:** Calibration points used for divergence time estimation in BEAST. The offset values from the BEAUTI settings column correspond to assigned fossil ages

Fossil name	Clade constrained	Plant organs and synapomorphies	Primary reference	BEAUTI settings
*Machaerium*	Stem of Tribe Dalbergieae (Leguminosae)	Fossil leaflets. Strong marginal vein, poorly organized higher order venation, numerous closely spaced craspedodromous secondary veins, and epidermal cell structure are diagnostic characters for *Machaerium* (Tribe Dalbergieae)	(Herendeen, Crepet, & Dilcher, 1992)	Offset = 40, Mean = 1.0, *SD* = 0.5
*Fraxinus excelsior*	Crown of family Oleaceae	Fruit fossils. Winged (samara type) fruit that resembles *Fraxinus* in peduncle, vein structure and shape, and position of seed	(Jung & Lee, 2009)	Offset = 5.33, Mean = 1.0, *SD* = 0.5 (as used in Magallón et al., 2015)
*Sabalites carolinensis*	Stem of Tribe Cryosophileae (Subfamily Coryphoideae, Arecaceae)	Leaf fossil. Oldest known palm fossil assignable to Subfamily Coryphoideae with costapalmate leaf	(Dransfield et al., 2008)	Offset = 86.7, Mean = 1.7, *SD* = 0.3 (as used in Bacon et al., 2012)
*Micrantheum spinyspora*	Stem of family Picrodendraceae	Pollen fossils	(Christophel, Harris, & Syber, 1987)	Offset = 35.55, Mean = 1.0, *SD* = 0.5
*Acalypha*	Stem of tribes Adelieae and Pluketenieae (Subfamily Acalyphoideae, Euphorbiaceae	Pollen fossils. Diagnostic characters of Acalyphoideae include pollen and pores of small size; sculpture punctate–reticulate; thick nexine and separate from sexine around pore, making sexine in the aperture protruding in a fastigium‐like chamber	(Sun et al., 1989)	Offset = 61.0, Mean = 1.0, *SD* = 0.5 (as used in Davis, Webb, Wurdack, Jaramillo, & Donoghue, 2005)
*Solanispermum reniforme*	Stem of family Solanaceae	Fossil seeds; one of the earliest fossils assigned to Solanaceae	(Chandler, 1962)	Offset = 47.0, Mean = 1.0, *SD* of 0.5 (as used in Martínez‐Millán, 2010)
*Trithrinax dominicana,*	Stem of genus *Trithrinax* (Arecaceae)	Flower fossils. Stamen filaments exerted and tips bent inwards are diagnostic characters for *Trithrinax*	(Poinar, 2002)	Offset = 24.5, Mean = 1.0, *SD* of 0.5
*Prosopis linearifolia*	Stem of *Umtiza* clade (Fabaceae)	Fossil leaves. Mix of pinnate and bipinnate leaves. Leaflets linear and asymmetric. Terminal group of three pinnae in a single bipinnate leaf, rising from a sessile terminal pinna. These diagnostic characters are associated with *Arcoa* (*Umtiza* clade)	(Herendeen, Lewis, & Bruneau, 2003)	Offset = 34.0, Mean = 1.0, *SD* of 0.5 (as used in Lavin, Herendeen, & Wojciechowski, 2005)
	Stem of Angiosperms	Secondary calibration point	(Silvestro et al., 2015)	Laplace prior distribution, Offset = 143.7, μ = 1.0, scale = 4.36
	Stem of Spermatophyta	Secondary calibration point	(Silvestro et al., 2015)	Gamma prior distribution, Offset = 366.0, Mean = 1.0 *SD* = 0.5

The BI analysis was run on Westgrid's “*Parallel”* cluster (Compute Canada Services) for a total of 891 million generations of Markov chain Monte Carlo (MCMC), with parameters sampled every 30,000 generations and discarded 25% as burn‐in using TREEANNOTATOR 2.3.1 (Bouckaert et al., 2014). Availability of time at the cluster determined the number of generations. The resulting log file was checked in TRACER v1.6 (Rambaut, Suchard, Xie, & Drummond, 2014) to assess convergence using effective sample size (ESS) values, and the log likelihood versus the generation number plots. The final number of trees used to generate the maximum clade credibility (MCC) tree was 19,079.

### 2.4 Ancestral area estimation

**Figure 1:**
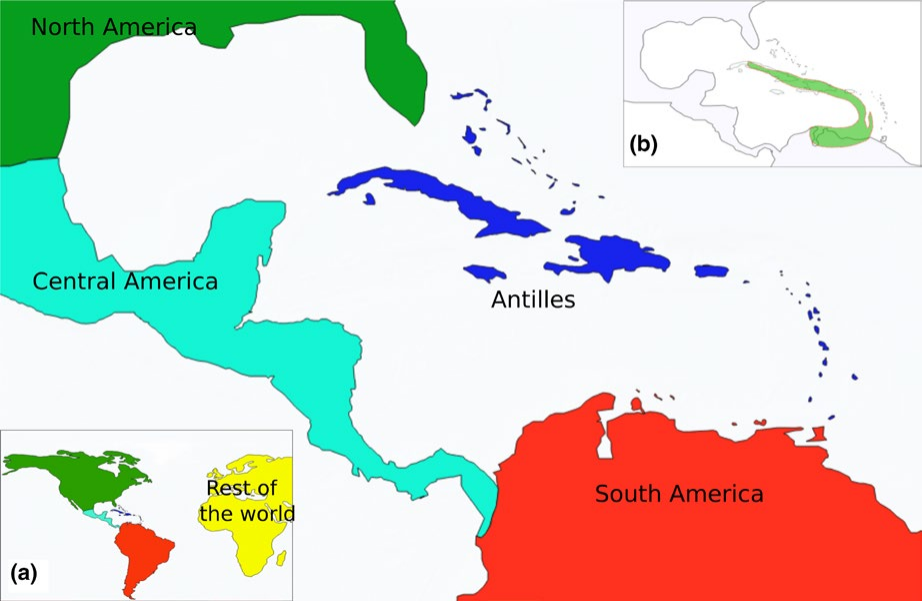
Operational biogeographic areas used in this study. *Inset*s: (a) the five operational areas. (b) The GAARlandia land bridge hypothesized to have existed between 33 and 35 Ma (modified from Iturralde‐Vinent & MacPhee, 1999)

We used the ML method implemented in the R package BioGeoBears v.0.2.1 (Matzke, 2013) to estimate the evolution of geographic ranges in endemic genera. BioGeoBears allows estimating the ancestral range of taxa using several inference models, such as dispersal, extinction, and cladogenesis (DEC, Ree, Moore, Webb, & Donoghue, 2005; Ree & Smith, 2008), dispersal–vicariance (DIVA, Ronquist, 1997), and Bayesian biogeographic inference (BayArea, Landis, Matzke, Moore, & Huelsenbeck, 2013). BioGeoBears requires an ultrametric tree (we used the MCC tree from BEAST) and a matrix of geographic distributions in presence–absence format. As BioGeoBears requires positive branch lengths (Matzke, 2013), we manually edited the only negative branch length by adding 0.3 nucleotide substitution per site. To prepare the presence–absence matrix, we obtained species distributions from the Global Biodiversity Information Facility (GBIF, https://www.gbif.org/, accessed 12 June, 2015). We defined five biogeographic operational areas: (A) Antilles; (B) Central America; (C) South America; (D) North America; and (E) rest of the world (Figure 1). Species distributions were coded using the R implementation in the software package SpeciesGeoCoder v.1.0‐4 (Töpel et al., 2016). The output presence–absence matrix was visually inspected and corrected manually for erroneous assignments.

We first ran a null analysis with no time frames and equal rates of dispersal among operational areas for each of the six biogeographic models (DEC; DEC*j*; BAYEAREALIKE; BAYAREALIKE*j*; DIVALIKE; DIVALIKE*j*). A second and more complex stratified model was run in order to reflect more realistically the paleogeographic framework of the Caribbean allowing different dispersal rates among operational areas at six different time frames: (1) 0–15 Ma; (2) 15–33 Ma; (3) 33–35 Ma; (4) 35–50 Ma; (5) 50–130 Ma; and (6) 130–378 Ma. Equal dispersal probabilities between regions were scaled from 0 (e.g., when areas were not yet formed) to 1 (e.g., when a land bridge or continuous landmass is proposed to have connected operational areas). We used intermediate values (i.e., 0.01, 0.1, and 0.5) to constrain dispersal events reflecting the presumed biotic connectivity between areas. To test the hypothesized directionality of dispersal events from south to north (Bacon et al., 2015; Weir et al., 2009), a third complex stratified model was run using unequal dispersal probabilities for the period 0–15 Ma. We allowed an extra 0.25 with respect to the previous complex model for the dispersal probabilities from SA to the Antilles and from SA to NA. The complete dispersal matrices used in the ancestral area reconstruction analysis are shown in Table 3 and the detailed explanation of the paleogeographic context under each time frame is as follows:

**Table 3:** Dispersal matrices used in BioGeoBears for complex biogeographic modeling

Complex model 1 and 2														
AN	CA	NA	RW	SA	AN	CA	NA	RW	SA	AN	CA	NA	RW	SA
0–15 Ma					15–33 Ma					33–35 Ma				
1	0.5	0.5	0.1	0.5	1	0.5	0.5	0.1	0.5	1	0.5	0.5	0.1	1
0.5	1	1	0.1	1	0.5	1	1	0.1	1	0.5	1	1	0.1	1
0.5	1	1	0.1	0.5	0.5	1	1	0.1	0.1	0.5	1	1	0.1	0.1
0.1	0.1	0.1	1	0.1	0.1	0.1	0.1	1	0.1	0.1	0.1	0.1	1	0.1
0.5 (0.75)	1	0.5 (0.75)	0.1	1	0.5	1	0.1	0.1	1	1	1	0.1	0.1	1

35–50 Ma					50–130 Ma					130–378 Ma				
1	0.5	0.5	0.01	0.5	1	0.01	0.1	0.01	0.1	1	0	0	0	0
0.5	1	0.1	0.01	0.1	0.01	1	0.01	0.01	0.01	0	1	0.01	0.01	0.01
0.5	0.1	1	0.1	0.1	0.1	0.01	1	0.01	0.01	0	0.01	1	0.01	0.01
0.01	0.01	0.1	1	0.1	0.01	0.01	0.01	1	0.01	0	0.01	0.01	1	0.01
0.5	0.1	0.1	0.1	1	0.1	0.01	0.01	0.01	1	0	0.01	0.01	0.01	1

Complex model number 1 accounts for equal dispersal probabilities in both directions between areas. Complex model number 2 is identical as complex model 1 except for the dispersal probabilities from South America to the Antilles and from South America to North America which are increased by 0.25 in model 2 (values shown within parentheses), thus favoring dispersal South to North for the 0–15 Ma period.

1. 0–15 Ma: From the Middle Eocene to the Holocene, landmasses had approximately occupied their current position. The Central American Seaway between South America and the Panama Bloc was fully closed by 15–13 Ma (Jaramillo et al., 2017; Montes et al., 2015) facilitating biotic interchange between North and South America as shown for wide range of taxonomic groups in Bacon et al. (2015). We therefore set up a dispersal constraint of 0.5 between North and South America and gave the maximum dispersal score of 1 between possible dispersal events between Central and North America and between Central and South America reflecting connectivity between landmasses (De Baets, Antonelli, & Donoghue, 2016). During this period, the Antilles were already above water; therefore, dispersal from/to the Antilles and the surrounding landmasses was possible; we therefore set a constraint of 0.5 for dispersal from/to the Antilles and North America, Central America, and South America, and a minimal constraint of 0.1 from/to the rest of the world. Northern Hemisphere landmasses were at least partially connected through land bridges that increased the connectivity among regions. The Beringia land bridge connected Eurasia to North America and was interrupted around ca. 5.5 Ma (Gladenkov, Oleinik, Marincovich, & Barinov, 2002). The North Atlantic land bridge connecting Europe to North America was hypothesized to have existed between the regions up to the Eocene (Tiffney, 1985); however, studies based on ocean microfauna and ocean circulation patterns suggest that the land bridge might have existed until as late as 15 Ma (Poole & Vorren, 1993; Schnitker, 1980). Therefore, we set a minimal constraint of 0.1 to reflect potential dispersal from/to North America and the rest of the world, and the same 0.1 constraint from/to South America and the rest of the world for potential long distance dispersal events;
2. 15–33 Ma: We reduced the dispersal probabilities from/to North and South America to 0.1 in order to reflect the preclosure of the Panama Isthmus (Montes et al., 2015);
3. 33–35 Ma: For this time frame, we kept the same dispersal probabilities as in time frame 1 but allowed a higher dispersal probability of 1 from/to South America and the Antilles to reflect the hypothesized GAARlandia land bridge;
4. 35–50 Ma: From Early to Late Eocene. We set a dispersal probability of 0.5 from/to Antilles and North America, South America, and Central America. As Central America was not fully formed, we set a probability of 0.1 for potential dispersal events from/to Central and North America, and Central and South America. We also set a probability of 0.01 from/to Central America and the rest of the world. A probability of 0.1 was given for dispersal events from/to North and South America;
5. 50–130 Ma: From Late Eocene to Lower Cretaceous. Central America was not fully formed, restricting the possibility of dispersal between North America and South America (Montes et al., 2015). To reflect potential long distance dispersal events, we set a constraint of 0.01 from/to North and South America, and also from/to North America and the rest of the world, and from/to South America and the rest of the world. We imposed a dispersal constraint of 0.01 for migrations from/to Central America and the Antilles and also 0.01 from/to Central America and the rest of the world. Same minimal dispersal probability of 0.01 from/to Central America and South America and from/to Central America and North America. Greater Antilles were above water, and Lesser Antilles started forming (Graham, 2003; Pindell & Kennan, 2009), thus, we imposed a minimal dispersal probability of 0.1 from/to Antilles to South and North America;
6. 130–378 Ma: From the Middle Devonian to Late Jurassic, landmasses were mostly conglomerated, and the Pangea supercontinent started to break up at about 200 Ma, forming Gondwana and Laurasia. Gondwana started to break up about 150 Ma. We reduced all dispersal constrains in this time frame to 0.01 as the lower bound of this time frame is contemporaneous to the estimated origin of Angiosperms (Bell, Soltis, & Soltis, 2010; Silvestro, Cascales‐Miñana, Bacon, & Antonelli, 2015), and it is prior to the origin of Zamiaceae (Salas‐Leiva et al., 2013). We did not allow any dispersal event from/to the Antilles as those islands had not formed yet.

### 2.5 A compilation of independent evolutionary and biogeographic studies on a subset of Caribbean endemic genera

An uneven and/or limited taxon sampling and lack of phylogenetic resolution resulting from a few sampled genes can bias estimates of divergence times and ancestral areas in the broad‐scale dated phylogenetic analysis (Linder, Hardy, & Rutschmann, 2005; Pirie & Doyle, 2012). We therefore conducted a second approach to contrast and validate our broad‐scale results using multiple independently dated phylogenetic trees. We compiled crown and stem ages from published trees that comprised the Caribbean endemic genera included in the broad‐scale analysis. We obtained information for 24 of the 41 endemic genera. Six of these studies (covering 11 endemic genera) investigated the ancestral areas for such endemic genera (Table 4).

**Table 4:** Compilation of independent dated phylogenies from the literature. Crown and stem ages in millions of years (Ma)

Endemic genus	Crown age in Ma	Stem age in Ma	Ancestral area estimation	References
*Acidoton*	1.7	2.6	SA	(Cervantes et al., 2016)
*Anacaona*	13	17	SA	(Schaefer et al., 2009)
*Arcoa*	34			(Lavin et al., 2005)
*Bonania*	41.6	46.39	MX, SA, MS	(Cervantes et al., 2016)
*Broughtonia*	15.68	20.74		(Sosa et al., 2016)
*Brya*	41.9	47.2		(Lavin et al., 2005)
*Cubanola*	19.2	47.3	AN	(Antonelli et al., 2009)
*Dilomilis*	16.01	46.72		(Sosa et al., 2016)
*Ditta*	95	105		(van Ee et al., 2008)
*Domingoa*	19.34	20.9		(Sosa et al., 2016)
*Hebestigma*	38.1	48.3		(Lavin et al., 2003)
*Hemithrinax*	6.99	17.54	AN	(Cano et al., unpublished data)
*Leptocereus*	2.8	4.8	SA	(Hernández‐Hernández et al., 2014)
*Lasiocroton*	1.17	10.76	AN	(Cervantes et al., 2016)
*Leucocroton*	5.27	10.76	AN	(Cervantes et al., 2016)
*Microcycas*	36.5	60.32	AF‐CA	(Salas‐Leiva et al., 2013)
*Neocogniauxia*	16.01	46.72		(Sosa et al., 2016)
*Penelopeia*	13	17	SA	(Schaefer et al., 2009)
*Pictetia*	14.5	45.6		(Lavin et al., 2005)
*Poitea*	9.2	16.4		(Lavin, Wojciechowski, et al., 2001)
*Psychilis*	15.68	20.74		(Sosa et al., 2016)
*Quisqueya*	15.68	20.74		(Sosa et al., 2016)
*Tetramicra*	15.68	20.74		(Sosa et al., 2016)
*Zombia*	3.75	21.7	AN	(Cano et al., unpublished data)

Ancestral area abbreviations: SA, South American ancestor; AN, Antillean ancestor; AF‐CA, African Caribbean ancestor; MX, Mexico; and MS, Mesoamerica.

## 3 RESULTS

### 3.1 Phylogenetic reconstruction and molecular dating

**Figure 2:**
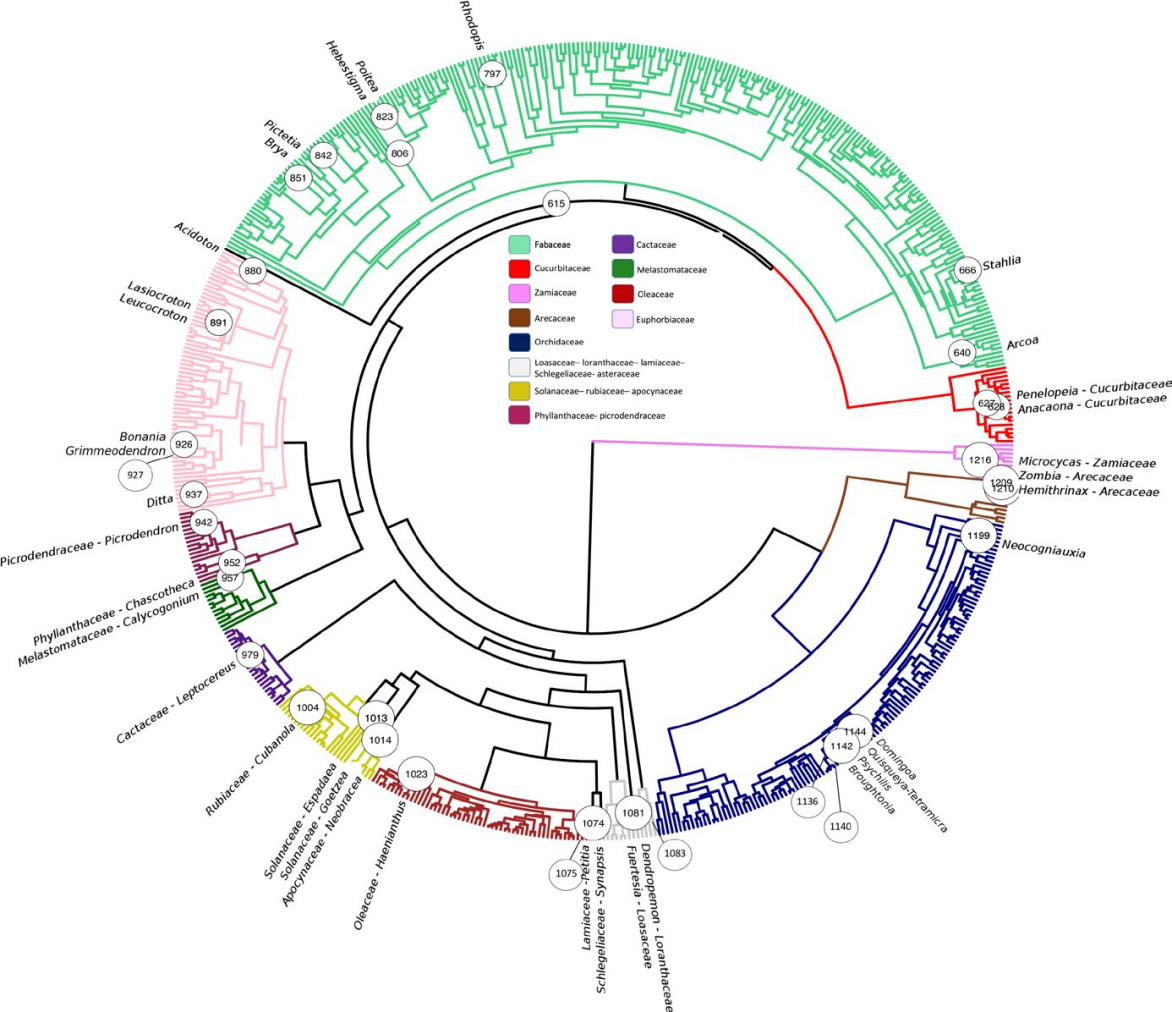
Phylogenetic relationships of plant families obtained from the Bayesian dating analysis (maximum clade credibility tree). Numbered circles indicate node number that subtends each Caribbean endemic genus or clade (same as in Table 5). Families within a clade represented in this study by a small number of taxa have been lumped into one color

The ML and BI analyses recovered congruent tree topologies for the higher relationships of taxa. Figure 2 shows the phylogenetic relationships among families and the distribution of endemic genera across the BI tree. ESS values were above 200, except for the treeLikelihood (ESS of 103), and the *Trithrinax* and Solanaceae calibration points (ESS of 39 and 23, respectively).

Most family relationships were congruent with the latest Apg (2009). In one exception, the BI, but not the ML analysis, recovered a clade of 15 Euphorbiaceae species within the Orchidaceae clade, which we therefore removed from the tree using the “drop.tip ()” function in the R package “ape” (Paradis, Claude, & Strimmer, 2004). Consequently, the endemic genera *Moacroton* and *Acidocroton* were removed from the dated tree and will not be further discussed. In addition, due to our taxon sampling criteria and DNA marker selection, some clades containing endemic genera had very few (<3) species, did not include the sister genus, or the suprageneric rank was poorly represented (Table 1). Therefore, divergence times and ancestral areas for the endemic genera *Doerpfeldia*,* Espadaea*,* Fuertesia*,* Goetzea*,* Haenianthus*,* Petitia,* and *Synapsis* could not be estimated accurately, and results are not shown. After these exclusions, the total number of endemic genera for which we present results is 32.

**Figure 3:**
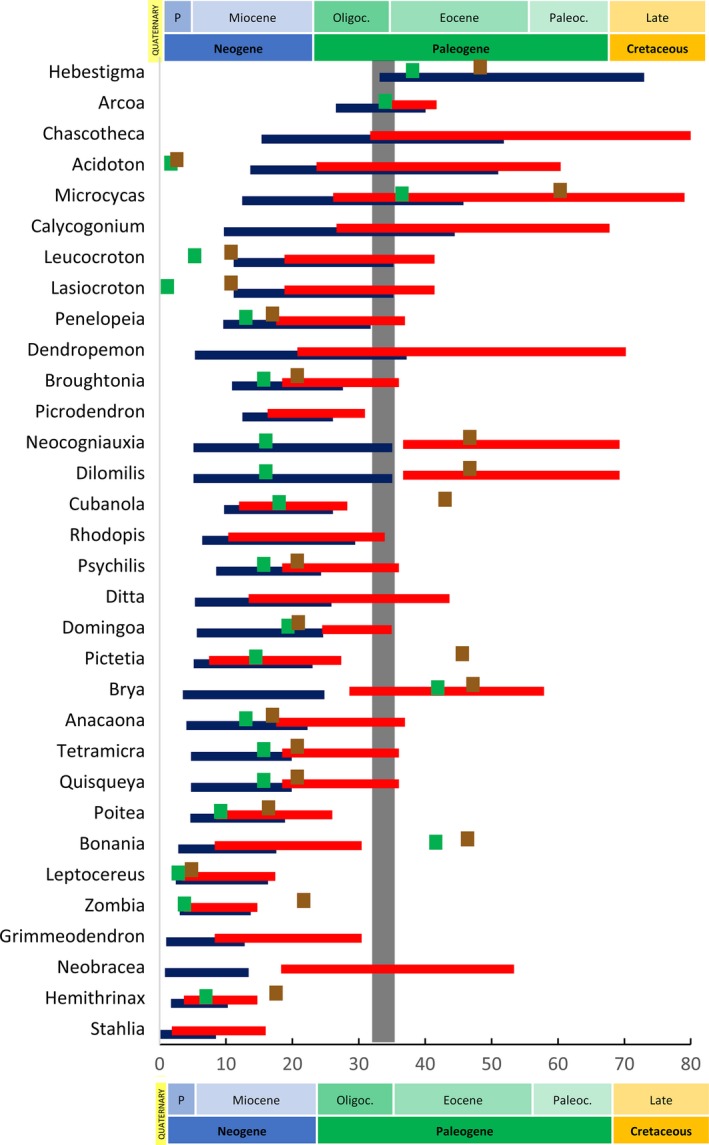
Bayesian divergence times of Caribbean endemic genera ordered by age. Blue and red bars correspond to the 95% HPD for the crown and stem node ages, respectively, obtained in our broad‐scale analysis. Green and brown squares represent crown and stem ages, respectively, obtained from the literature. Gray vertical band indicates the GAARlandia time frame (33–35 Ma). Geological timescale according to the International Commission on Stratigraphy (v2016/04; Cohen et al., 2013). Pliocene is abbreviated as P, the Oligocene as Oligoc., and the Paleocene as Paleoc. Note the 95% HPD for the stem of *Hebestigma* (88.57–123.62 Ma) and the mean crown (95 Ma) and stem (105 Ma) ages of *Ditta* are not shown in the figure because they fall outside the geological scale

The Bayesian dating analysis showed that divergence between Angiosperms and Gymnosperms occurred at 370 Ma ([95% HPD (higher posterior density) 366–374 Ma]). The mean crown age for the Angiosperms was estimated at 191 Ma (95% HPD 162–220 Ma), and 50.8 Ma (95% HPD 26.2–79.1) for the Zamiaceae. Mean crown ages of endemic genera dated from the Early Eocene [*Hebestigma*, Leguminosae: 53.1 (95% HPD 33.1–73.0) Ma] to the Pliocene [*Stahlia*, Leguminosae: 3.40 (95% HPD 0.0078–8.50) Ma], whereas mean stem ages ranged from the Late Cretaceous [*Hebestigma*, Leguminosae: 106 (95% HPD 88.6–123) Ma] to the Middle–Late Miocene [*Stahlia*, Leguminosae: 8.64 (95% HPD 1.86–15.9) Ma]. Eleven of the 32 endemic genera had stem and crown node 95% HPD ages younger than the GAARlandia time frame (<33 Ma), while 22 genera had stem and/or crown 95% HPD ages during the hypothesized land bridge (Figure 3). *Hebestigma* was probably the only genus that diverged before GAARlandia as the lowest 95% HPD bound of its crown age was estimated at 33 Ma. The mean crown ages of three endemic genera occurred within the GAARlandia time frame (*Acidoton* [Euphorbiaceae] at 31.8 Ma; *Arcoa* [Leguminosae] at 34.1 Ma; and *Chacotheca* [Phyllanthaceae] at 32.9 Ma). The mean stem age of *Neobracea* [Apocynaceae, 35.6 Ma] also fell within GAARlandia. Divergence time estimations at crown and stem nodes for endemic genera can be found in Table 5 (see also Figs S1 and S2 for BEAST MCC tree and node numbers in MCC tree). The four‐gene concatenated matrix, and BEAST MCC tree are available in Dryad (http://dx.doi.org/10.5061/dryad.gq93s).

**Table 5:** Divergence times resulting from a Bayesian dating analysis in BEAST at crown and stem nodes and ancestral area reconstruction for each genus showing the most likely ancestral area based on the Complex 1 DEC*j* model

Endemic genus	Crown node number	Mean ages at crown nodes in Ma (95% HPD)	Stem node number	Mean ages at stem nodes in Ma (95% HPD)	Ancestral Reconstruction probabilities (at stem nodes)
*Acidoton*	880	31.74 (13.65–50.99)	879	42.49 (23.65–60.72)	RW 0.38
*Anacaona*	628	12.71 (4.05–22.29)	627	27.67 (17.54–36.97)	AN 0.65
*Arcoa*	640	34.09 (26.56–40.05)	637	38.24 (34.68–41.72)	RW 0.80
*Bonania*	926	9.92 (2.81–17.60)	925	19.41 (8.32–30.44)	AN 0.19; ANNA 0.16*
*Broughtonia*	1,136	19.42 (10.91–27.63)	1,135	26.73 (18.45–36.05)	AN 0.83*
*Brya*	851	13.72 (3.52–24.82)	850	44.17 (28.59–57.89)	SA 0.94
*Calycogonium*	957	26.81 (9.67–44.44)	956	47.27 (26.64–67.79)	SA 0.53
*Chascotheca*	952	32.86 (15.37–52.74)	951	56.43 (31.72–80.80)	RW0.26; ANRW 0.22
*Cubanola*	1,004	17.65 (9.73–26.12)	1,002	20.25 (11.98–28.28)	AN 0.97
*Dendropemon*	1,083	20.45 (5.34–37.22)	1,082	44.19 (20.76–70.21)	CA 0.65
*Dilomilis*	1,199	18.74 (5.10–35.04)	1,111	52.80 (36.69–69.27)	AN 0.93*
*Ditta*	937	15.09 (5.32–25.90)	936	28.50 (13.42–43.68)	RW 0.80
*Domingoa*	1,144	15.009 (5.61–24.63)	1,116	30.67 (24.48–34.96)	AN 0.57*
*Grimmeodendron*	927	6.61 (0.99–12.81)	925	19.41 (8.32–30.44)	AN 0.19; ANNA 0.16*
*Hebestigma*	806	53.12 (33.13–72.98)	716	105.48 (88.57–123.62)	SA 0.29
*Hemithrinax*	1,210	6.07 (1.71–10.28)	1,209	9.35 (3.68–14.72)	AN 0.98
*Leptocereus*	979	9.07 (2.43–16.32)	978	11.12 (3.35–17.42)	SA 0.89
*Lasiocroton*	891	22.59 (11.16–35.23)	890	28.85 (18.81–41.40)	CA 0.54*
*Leucocroton*	891	22.59 (11.16–35.23)	890	28.85 (18.81–41.40)	CA 0.54*
*Microcycas*	1,216	28.23 (12.43–45.78)	1,215	51 (26.15–79.07)	AN 0.95
*Neobracea*	1,015	6.49 (0.81–13.42)	1,014	35.45 (18.29–53.39)	ANRW 0.61
*Neocogniauxia*	1199	18.74 (5.10–35.04)	1111	52.80 (36.69–69.27)	AN 0.93*
*Penelopeia*	627	20.91 (9.58–31.80)	626	27.67 (17.54–36.97)	CA 0.39
*Picrodendron*	942	19.13 (12.37–26.11)	941	23.93 (16.27–30.93)	RW 0.48
*Pictetia*	842	13.96 (5.15–23.03)	841	17.67 (7.47–27.37)	AN 0.45
*Poitea*	826	11.50 (4.63–18.89)	823	17.10 (8.50–26.01)	AN 0.25; ANCA 0.21
*Psychilis*	1,140	15.93 (8.51–24.31)	1,135	26.73 (18.45–36.05)	AN 0.83*
*Quisqueya*	1,142	12.08 (4.73–19.92)	1,135	26.73 (18.45–36.05)	AN 0.83*
*Rhodopis*	797	17.47 (6.42–29.48)	796	21.89 (10.33–33.92)	SA 0.14
*Stahlia*	666	3.40 (0.0078–8.50)	665	8.64 (1.86–15.97)	CA 0.67
*Tetramicra*	1,142	12.08 (4.73–19.92)	1,135	26.73 (18.45–36.05)	AN 0.83*
*Zombia*	1,209	8.13 (3.03–13.74)	1,208	9.35 (3.68–14.72)	AN 0.79

Asterisks indicate that the genus is part of an endemic clade (*). SA, South America; CA, Central America; NA, North America; AN, Antilles; RW, rest of the world.

### 3.2 Ancestral area estimation

The likelihood values for the null and the two complex stratified models can be found in Table 6 for the 18 models ran in BioGeoBears. Model selection did not support the hypothesized directionality of dispersal from south to north (complex model 2). The DEC*j* model from the complex model 1 (with founder effect, time stratification and symmetrical dispersal constraints) was selected as the most appropriate for our data set (Lnl = −1221.2 and AIC = 2,448.3), while the second‐best model was the DEC*j* from the complex model 2 (Lnl = −1235.1 and AIC = 2,476.3).

**Table 6:** Biogeographic model testing in BioGeoBears

	LnL	# params	*d*	*e*	*j*	AIC	AIC wt
Null model							
BAYAREALIKE	−1,480.99	2	0.00389	0.019351951	0	2,965.992259	3.63E‐96
BAYAREALIKEj	−1,260.30	3	0.00228	0.000617994	0.046701184	2,526.614158	0.933204519
DEC	−1,318.92	2	0.00449	1.00E‐12	0	2,641.850873	8.84E‐26
DECj	−1,262.94	3	0.00356	1.00E‐12	0.028423902	2,531.888136	0.066795481
DIVALIKE	−1,330.90	2	0.00541	1.00E‐12	0	2,665.809754	5.55E‐31
DIVALIKEj	−1,294.20	3	0.00428	1.08E‐09	0.022922813	2,594.403385	1.78E‐15
Complex model 1							
BAYAREALIKE	−1,465.10	2	0.01131	0.016440112	0	2,934.215946	3.10E‐106
BAYAREALIKEj	−1,235.58	3	0.00847	0.000807486	0.126630982	2,477.169101	5.48E‐07
DEC	−1,288.10	2	0.01513	0.000614288	0	2,580.218724	2.30E‐29
DECj^a^	−1,221.16	3	0.01144	4.87E‐05	0.115873802	2,448.333573	0.999999452
DIVALIKE	−1,295.18	2	0.018188	0.000809571	0	2,594.36264	1.95E‐32
DIVALIKEj	−1,272.81	3	0.014687	0.000472286	0.020573461	2,551.631877	3.71E‐23
Complex model 2							
BAYAREALIKE	−1,468.83	2	0.01044	0.016429327	0	2,941.666889	8.73E‐102
BAYAREALIKEj	−1,243.37	3	0.00805	0.000673963	0.122983955	2,492.751708	0.00026405
DEC	−1,296.90	2	0.01430	0.00060304	0	2,597.809953	4.06E‐27
DECj^b^	−1,235.13	3	0.01109	5.33E‐05	0.063058035	2,476.273491	0.99973595
DIVALIKE	−1,304.63	2	0.01719	0.000805488	0	2,613.262891	1.79E–30
DIVALIKEj	−1,281.03	3	0.01505	0.000812842	0.019658281	2,568.078678	1.16E‐20

LnL, log likelihood; # params, number of parameters; *d*, dispersal rate per million years along branches; *e*, extinction rate per million years along branches; *j*, founder event speciation weighted per speciation event; AIC, Akaike Information Criterion; AIC wt, relative likelihood for each model. Best and second‐best model according to AIC values marked as “a” and “b,” respectively.

The complete ancestral area estimation using the DEC*j* model (complex model 1) is shown in Fig. S3. To plot the most likely ancestral distribution of endemic genera, we selected their corresponding stem nodes. When an endemic genus was sister to another endemic genus, we treated both genera as a unit (i.e., endemic clade) and selected the stem node of the endemic clade to plot the results. This was the case of *Lasiocroton*–*Leucocroton, Dilomilis*–*Neocogniauxia*, and *Broughtonia*–*Psychilis*–*Quisqueya*–*Tetramicra*. We considered *Bonania*–*Grimmeodendron* also as an endemic clade, because the sister relationship of *Grimmeodendron eglandulosum* and *Sebastiania bilocularis* (a nonendemic species) was not well supported in the tree [posterior probability (*pp*) of .43], and *Grimmeodendron–Sebastiana* was sister to the endemic genus *Bonania* with strong support (*pp *= .99).

**Figure 4:**
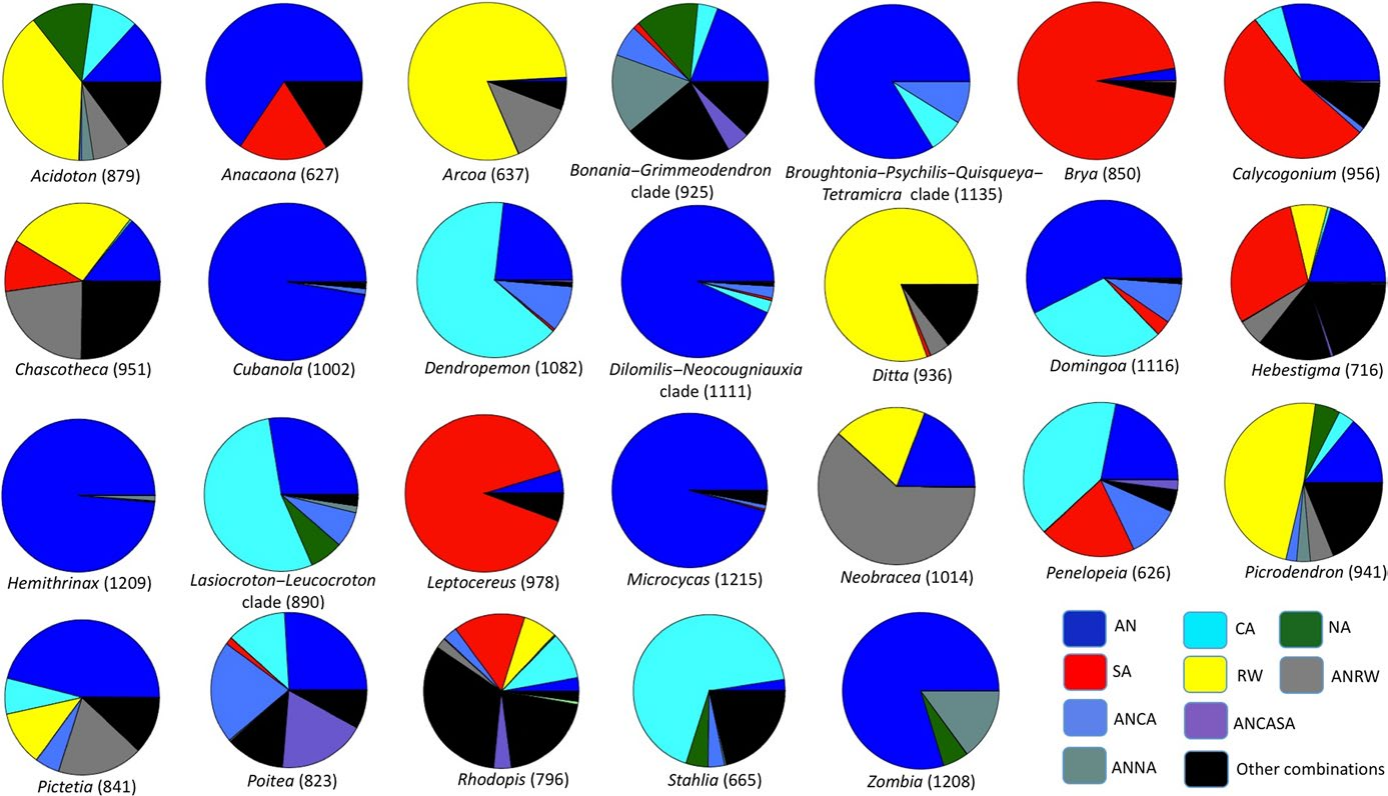
Ancestral area estimation for Caribbean endemic genera or clades based on the DEC *j* Complex model 1. Each pie chart contains the likelihood percentage for each estimated area per genus or clade. Numbers in parenthesis are selected nodes that subtend each endemic genus or clade in the tree and that were used for plotting results (same as in Table 5). Ancestors distributed in Antilles and Central America are abbreviated as ANCA; ancestors distributed in Antilles and rest of the world are abbreviated as ANRW; ancestors distributed in Antilles and North America are abbreviated as ANNA; and ancestors distributed in Antilles, Central America, and South America are abbreviated as ANCASA

Our results show that nine endemic genera or clades and their sister groups had ancestors distributed in the Antilles (i.e., highest probability values for the Antilles, Figure 4). This corresponds to a total of 16 of the 32 sampled endemic genera. Four endemic genera or clades (*Lasiocroton*–*Leucocroton*,* Penelopeia, Stahlia,* and *Dendropemon*) colonized the Antilles from CA. Five genera (*Brya*,* Calycogonium*,* Hebestigma*,* Leptocereus,* and *Rhodopis*) had ancestors distributed in SA, and five genera (*Acidoton*,* Arcoa*,* Chascotheca*,* Ditta,* and *Picrodendron*) had ancestors widely distributed in the rest of the world. The ancestral area of *Neobracea* was estimated in the Antilles and rest of the world (Figure 4). Furthermore, none of the endemic genera surveyed had ancestors solely distributed in NA. Nine endemic genera or clades had highest area probabilities of <50%, illustrating the degree of uncertainty in the analysis (see Table 5 for ancestral reconstruction probabilities for each genus).

Our analysis also recovered ten instances in which continental taxa appeared nested within a Caribbean clade, suggesting potential island to mainland colonization. More specifically, we detected five colonization events from the Antilles to CA (in Orchidaceae, Leguminosae, Euphorbiaceae, Rubiaceae, and Arecaceae) and one to SA (in Cucurbitaceae). In three instances, the nested continental taxa had widespread distributions in continental America [in Zamiaceae and Phyllanthaceae (CA and SA), Rubiaceae (AN and CA), and Leguminosae (CA and NA)].

### 3.3 A compilation of independent evolutionary and biogeographic studies on a subset of Caribbean endemic genera

We found 10 studies that reported crown and stem ages for 24 endemic genera (Table 4). In these studies, divergence times ranged from 1.17 (in *Lasiocroton*) to 95 Ma (in *Ditta*) for the crown ages and from 2.6 (in *Acidoton*) to 105 Ma (in *Ditta*) for the stem ages. Five genera (*Acidoton*,* Bonania*,* Ditta*,* Lasiocroton*, and *Leucocroton*) had stem and crown ages outside the 95% HPD interval recovered in our broad‐scale analysis, the rest (18) had crown and/or stem ages inside our 95% HPD interval, and thus, we consider them in agreement with our broad‐scale approach.

A biogeographic origin was proposed across six studies for eleven endemic genera based on several methods including DEC, RASP, and DIVA‐GIS. Five genera were hypothesized to have reached the Antilles from SA (*Acidoton*,* Anacaona*,* Cubanola*,* Penelopeia,* and *Leptocereus*), while four had ancestors distributed in the Antilles (*Lasiocroton*,* Leucocroton*,* Hemithrinax,* and *Zombia*). The endemic cycad genus *Microcycas* was reported to have an African Caribbean ancestor, and an ancestor distributed in parts of Mexico, Mesoamerica, and SA was recovered for *Bonania* (Table 4).

## 4 DISCUSSION

The origin of endemic genera exhibited a mixed pattern of colonization from continental masses and in situ radiations within the islands, where all continental surrounding masses except for NA appeared to be sources for island colonization. Twenty‐two of the 32 genera had crown and/or stem node 95% HPD ages within the hypothesized GAARlandia time span. However, based on the range evolution analysis, we found no support for the hypothesized facilitative role of GAARlandia for SA colonizers, as crown and stem ages for endemic genera with ancestors distributed in SA did not fall within the GAARlandia period.

### 4.1 Oligocene to Miocene origin of Caribbean endemic plant genera

The age for the split between Angiosperms and Gymnosperms in Silvestro et al. (2015, 95% HPD 367.2–382.3 Ma) is congruent with the one we found in our study. The crown age we recovered for the Angiosperms was also concordant with earlier studies (Bell et al., 2010, 95; % HPD 167–199 Ma); Smith, Beaulieu, & Donoghue, 2010, 95; % HPD 182–257 Ma); Magallón, Hilu, & Quandt, 2013; 95% HPD 171.48–257.86 Ma) but older than that reported in Silvestro et al. (2015, 95% HPD 133.0–151.8 Ma) and Magallón, Gómez‐Acevedo, Sánchez‐Reyes, and Hernández‐ (2015, 95% HPD 136–139.95 Ma).

Our results showed that endemic plant genera of the Caribbean originated (mean stem to crown node ages) from the Late Cretaceous (ca. 105 Ma) until the Pliocene (ca. 3 Ma), a period during which the Caribbean islands reached their current position with respect to the surrounding continental masses. These divergence times support the perception that at least some endemic Caribbean biota reflects the ancient geological history of the archipelago (Ricklefs & Bermingham, 2008), and a model of continuous assembly of generic diversity in the Caribbean based on Dominican amber deposits from the early Eocene to early Miocene (Iturralde‐Vinent & MacPhee, 1996). Our inferred mean crown and stem ages were generally congruent with previous studies that have included endemic Caribbean genera, except for five genera in the Euphorbiaceae, for which the literature reports stem and crown ages outside our age confidence intervals (Figure 3). Divergence times for *Acidoton, Leucocroton*, and *Lasiocroton* estimated here were older than those reported in Cervantes, Fuentes, Gutiérrez, Magallón, and Borsch (2016). This is expected even for cases where fossil calibrations are correctly implemented in a molecular dating analysis (including their phylogenetic placement, age, and implementation). This is because fossils only provide minimum ages, and some fossils should just by chance be far too young in relation to the taxon they represent. By performing a single molecular dating analysis with a large supermatrix and several fossils for calibration, our results should reduce such stochastic errors and provide a more consistent estimation for all internal clade ages (see also Antonelli et al., 2017). The divergence time for *Ditta* inferred here was much younger than the estimate of van Ee et al. (2008). Their estimate should be taken with caution, however, as *Ditta* was sampled as part of the out‐group in a species‐level phylogeny focused on *Croton* subgenus *Moacroton* which belongs to a different tribe.

We found 22 of the 32 genera for which the 95% HPD ages at the stem and/or crown nodes overlap with the GAARlandia time frame. As divergence times alone cannot unequivocally support the GAARlandia hypothesis, we discuss below their relevance in light of the range evolution analysis.

### 4.2 Colonization from the continent and in situ speciation of Caribbean endemic plant genera

The Caribbean archipelago is considered to be sufficiently isolated from continental masses to allow allopatric divergence, but relatively close to maintain a dynamic island–continental interaction of biota (Ricklefs & Bermingham, 2008). As the Antilles is surrounded by continental landmasses, one might expect a great proportion of Caribbean ancestors to have occurred in them, and our results support this. About 32% of the endemic genera had ancestors distributed in continental America. Our results support overseas dispersal as an important factor to explain the distribution of endemic genera in the Antilles, in contrast to earlier works based on vicariance biogeography, which proposed that the Caribbean biota reflects the early geological history of the Proto‐Antilles arc (Rosen, 1975, 1985).

Our biogeographic analysis recovered CA, SA, and areas from the Old World as colonization sources for endemic plant genera in the Caribbean. Central American ancestors most probably reached the islands via the Central American Seaway, which is inferred by simulation models to have had a west‐to‐east direction prior to the closure of the Isthmus of Panama (Sepulchre et al., 2014). However, wind dispersal cannot be ruled out as it has been documented for sister genera (Cervantes et al., 2016; Renner, 2004), and hurricanes occur frequently in the region (Hedges, 2001). An exception to these dispersal modes (sea currents and wind) is the endemic genus *Dendropemon* (Loranthaceae) with a Central American ancestor and with seeds exclusively consumed by frugivorous birds (Kuijt, 2011). Our results of a Central American ancestor disagree with those reported in the literature in two cases. The first is the endemic clade *Lasiocroton*–*Leucocroton* (Euphorbiaceae), for which Jestrow, Gutiérrez, and Francisco‐Ortega (2012) and Cervantes et al. (2016) suggested an ancestor in eastern Cuba and the Antilles, respectively. The second is *Penelopeia* (Cucurbitaceae), for which Schaefer, Heibl, and Renner (2009) proposed a South American origin for the subfamily Cucurbitoideae. We attribute this disagreement to the differences in taxon sampling.

Despite floristic similarities between SA and the Caribbean at the genus level (Acevedo‐Rodríguez & Strong, 2008), we only found five endemic genera with South American ancestors. Of these five, the mean stem ages of *Leptocereus* and *Rhodopis* (11 and 21 Ma, respectively) were too young for GAARlandia to have acted as a dispersal route, and the evolution of *Hebestigma* was too old (Figure 3). Only *Brya* and *Calycogonium* could have used GAARlandia to colonize the Antilles from SA as the 95% HPD age at the stem and/or crown nodes fell within the land bridge's time span. The literature only reports a biogeographic analysis for *Leptocereus*, which agrees with our SA ancestral area result (Hernández‐Hernández, Brown, Schlumpberger, Eguiarte, & Magallón, 2014).

Our analyses revealed Old World ancestors for five endemic genera. For example, *Acidoton* (Euphorbiaceae) formed a clade with two North American species of *Tragia*, and this clade is sister to southeast Asian and African species. The ancestor of this clade could have used Northern Hemisphere corridors, or a trans‐Atlantic or trans‐Pacific dispersal to reach the American continent (Heads, 2008; Michalak, Zhang, & Renner, 2010; Wei et al., 2015). However, Cervantes et al. (2016) proposed a South American ancestor for *Acidoton,* which we also attribute to their different taxon sampling. We found no formal biogeographic analysis for any of the other four genera in the literature.

Our analysis recovered Antillean ancestors for 15 endemic genera within nine clades. For example, for *Microcycas*, the only gymnosperm endemic genus included in this study, Salas‐Leiva et al. (2013) proposed an African Caribbean ancestor for the clade *Stangeria*–*Zamia*–*Microcycas*. The fact that the African genus *Stangeria* is not present in our analyses might explain the disagreement between the Antillean ancestor we recovered for *Microcycas* and their study. These two biogeographic analyses do not support the hypothesis based on fossil evidence that *Microcycas* originated in continental America, reached Cuba, and then became extinct in the continent (Hermsen, Taylor, Taylor, & Stevenson, 2006). Antillean‐distributed ancestors were recovered for all endemic orchids (seven genera within three clades). For the genus *Bonania* (Euphorbiaceae), our results showed an Antillean and North American distributed ancestor, whereas Cervantes et al. (2016), who did not include the endemic sister genus *Grimmeodendron*, recovered an ancestor distributed in Mesoamerica, SA, and the Caribbean. Our finding of Antillean‐distributed ancestors for the endemic legume genera *Pictetia and Poitea* was not in agreement with that of Lavin, Wojciechowski, et al. (2001) who using a cladistic vicariance analysis hypothesized on a boreotropical origin for these two endemic genera. We attribute this disagreement to differences in taxon sampling and biogeographic analysis method. Our analysis recovered the Antilles as the most likely ancestral range for the two palm genera *Hemithrinax* and *Zombia* and for *Cubanola* (Rubiaceae) corroborating the results of Cano et al. (unpublished data), and Antonelli, Nylander, Persson, and Sanmartın (2009), respectively.

Our phylogenetic framework also identified nine instances for which Antillean taxa acted as source for continental taxa. This result is in line with Bellemain and Ricklefs (2008), which highlights the important and traditionally neglected role of islands as sources to colonize continental masses as seen for some terrestrial animals. Islands acting as reservoir of genetic diversity for the assemblage of continental floras have been reported for plants in other island systems (Andrus, Trusty, Santos‐Guerra, Jansen, & Francisco‐Ortega, 2004; Carine, Russell, Santos‐Guerra, & Francisco‐Ortega, 2004; Condamine, Leslie, & Antonelli, 2016; Patiño et al., 2015). Our results showed that all the operational areas defined in this study received immigrants from the islands. The time of these recolonizations ranges from Late Pleistocene to Early Oligocene (between 2 and 25 Ma). By then, most geological events that led to the current formation of the islands had taken place, rendering overwater, bird, or wind dispersal the most plausible explanation.

## 5 CONCLUSIONS

This is, to our knowledge, the first attempt to synthesize the evolutionary history of endemic plant lineages from the Caribbean. The high percentage of unsampled endemic genera in molecular phylogenies illustrates the knowledge gap of systematic and biogeographic studies on the Caribbean flora, and efforts should focus on sequencing more endemic taxa and their continental relatives. This would allow to further test our results of a mixture of primarily recent (Oligocene–Miocene) but also old (Paleocene–Eocene) lineages giving origin to the extant Caribbean endemic flora, and of predominantly Antillean ancestors of endemic genera. In addition, little is known about the reproductive biology of most seed plants endemic to the Caribbean region. This limited amount of natural history studies prevents us from generalizing on the dispersal patterns or mechanisms that ancestral lineages used to colonize and diversify in the Caribbean.

Our research does not support a role for GAARlandia as a colonization route to the Antilles for plants, and further geological evidence on the timing and geomorphology of this proposed land bridge awaits to be combined with molecular dating and biogeographic modeling approaches for a larger number of endemic lineages.

## CONFLICT OF INTEREST

None declared.

## AUTHOR CONTRIBUTIONS

M.E.N.B., J.R., and A.A. designed the study. M.E.N.B. compiled and analyzed the data. M.E.N.B. wrote the article with contributions from J.R. and A.A.
