# Supplementary figures and images for: Historical Biogeography of endemic seed plant genera in the Caribbean: Did GAARlandia play a role?
Source: Ecol Evol. 2017 Oct 24;7(23):10158–74. doi: 10.1002/ece3.3521 (PMC5723623; doi:10.1002/ece3.3521)

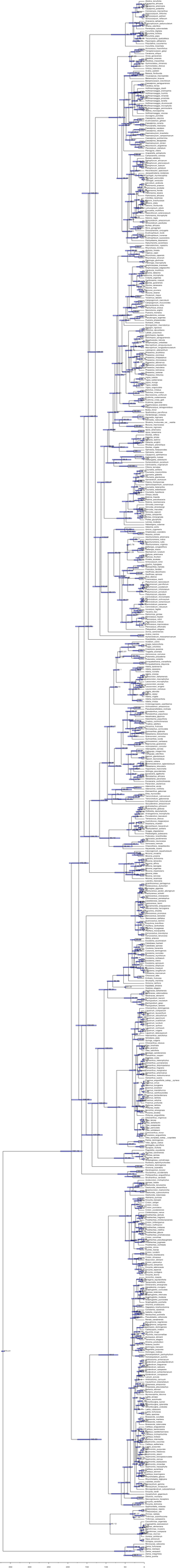

Supplement: Supplementary file 1 [file ECE3-7-10158-s001.pdf]

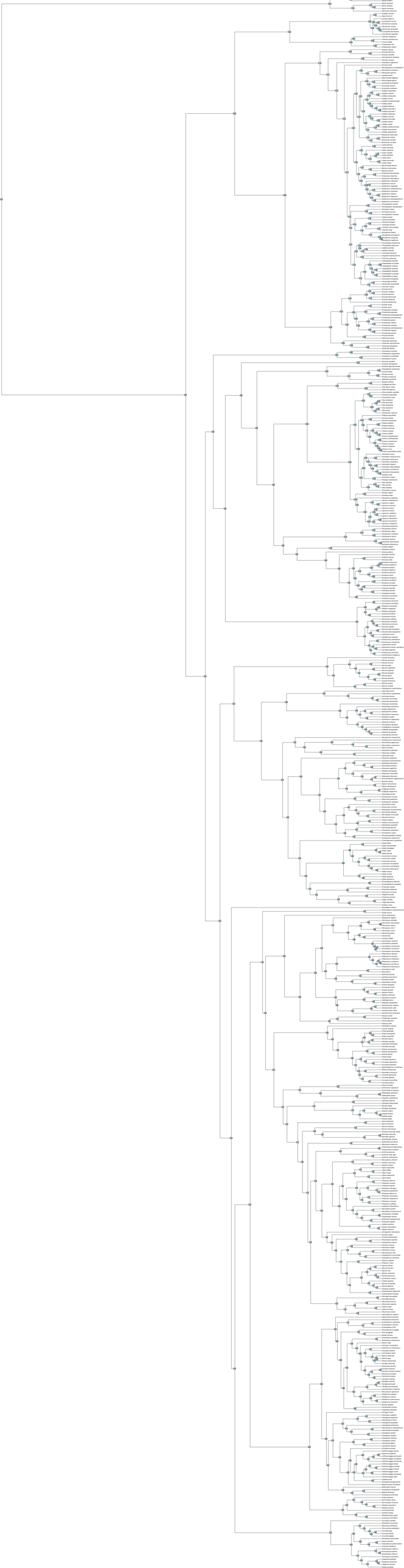

Supplement: Supplementary file 2 [file ECE3-7-10158-s002.pdf]
