# Supplementary material for: Historical Biogeography of endemic seed plant genera in the Caribbean: Did GAARlandia play a role?
Source: Ecol Evol. 2017 Oct 24;7(23):10158–74. doi: 10.1002/ece3.3521 (PMC5723623; doi:10.1002/ece3.3521)

LARGE_DEC+J_COMPLEX_1

ancstates: global optim, 5 areas max. d=0.0114; e=0; j=0.1159; LnL=−1221.17

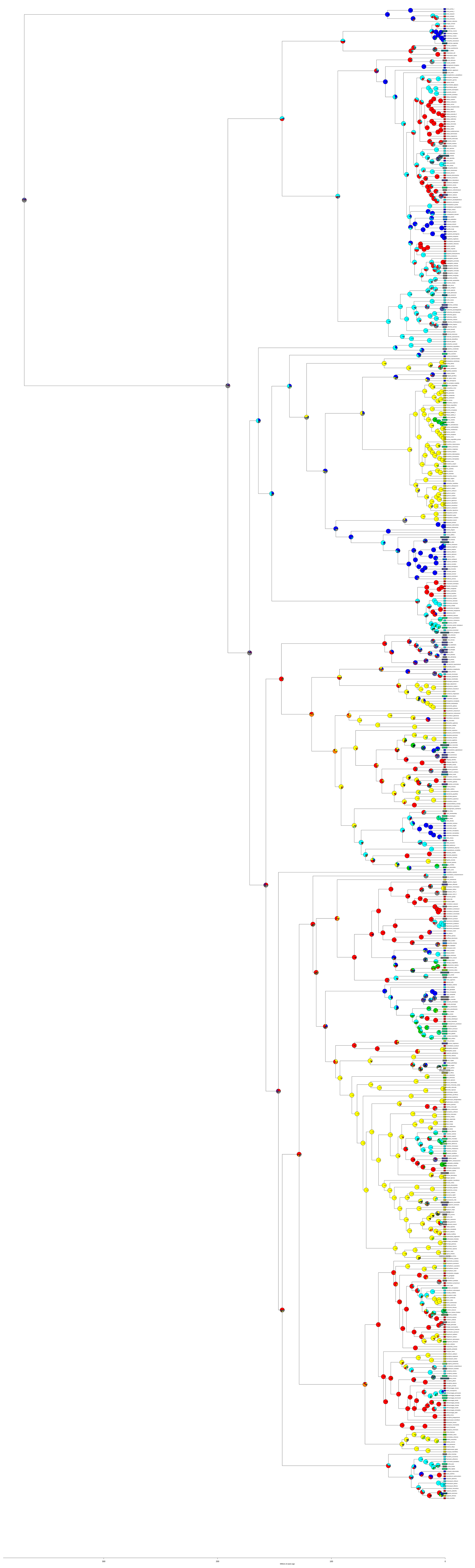

Supplement: Supplementary file 3 [file ECE3-7-10158-s003.pdf]
